# High Glucose Exacerbates TNF-*α*-Induced Proliferative Inhibition in Human Periodontal Ligament Stem Cells through Upregulation and Activation of TNF Receptor 1

**DOI:** 10.1155/2020/4910767

**Published:** 2020-02-05

**Authors:** Wenjun Zhu, Qihong Qiu, Haoyuan Luo, Fuping Zhang, Juan Wu, Xiaorui Zhu, Min Liang

**Affiliations:** ^1^Department of Periodontology, Guanghua School of Stomatology, Hospital of Stomatology, Sun Yat-sen University, Guangzhou 510055, China; ^2^Guangdong Provincial Key Laboratory of Stomatology, Guangzhou 510055, China; ^3^Department of Stomatology, Shenzhen Nanshan Hospital, Shenzhen 518000, China

## Abstract

**Objective:**

This research is aimed at investigating how high glucose affects the proliferation and apoptosis in periodontal ligament stem cells (PDLSCs) in the presence of TNF-*α*.

**Methods:**

PDLSCs obtained from periodontal healthy permanent teeth were treated under either high-glucose condition (30 mmol/L, G30 group) or normal glucose condition (5.6 mmol/L, G5.6 group) in the presence or absence of TNF-*α* (10 ng/ml) for 2 to 6 days. Cell proliferation and cell cycle were evaluated by CCK-8, EdU incorporation assay, and flow cytometry. Cell apoptosis was assessed by annexin V/PI staining. Protein expression was detected by western blotting. Cellular ROS expression was evaluated by CellROX labeling and flow cytometry. Specific antibodies targeting TNFR1 and TNFR2 were used to block TNF-*α* signaling. Vitamin C was also used to verify if the blockage of ROS can rescue PDLSCs in the presence of high glucose and TNF-*α*.

**Results:**

CCK-8 assay showed that high glucose exacerbated TNF-*α*-induced cell viability inhibition (57.0%, 85.2%, and 100% for the G30+TNF-*α* group, G5.6+TNF-*α* group, and control group, respectively) on day 6. High glucose increased protein expression of TNFR1 compared with the control group on day 2 (1.24-fold) and day 6 (1.26-fold). Blocking TNFR1 totally reversed the proliferative inhibition in G30+TNF-*α* group. The addition of vitamin C or TNFR1 antibody totally reversed the elevation of intracellular ROS expression caused by high glucose and TNF-*α*. Vitamin C partially restored cell proliferation in the presence of high glucose and TNF-*α*.

**Conclusion:**

High glucose exacerbates TNF-*α*-induced proliferative inhibition in human periodontal ligament stem cells through the upregulation and activation of TNF receptor 1. Inhibition of intracellular ROS expression by vitamin C partially rescues PDLSCs in terms of cell proliferation.

## 1. Introduction

Periodontitis, which is caused by a bacterial infection and exacerbated by host immunological reactions, may result in attachment loss, bone resorption, and eventually loss of teeth. Fortunately, periodontal ligament stem cells (PDLSCs) have shown some promise as seeds for regenerating periodontal tissues [1]. PDLSCs are a subgroup of mesenchymal stem cells in terms of phenotype (express stromal cell surface markers, including CD146, Stro-1, CD105, CD90, and CD73) and multilineage differentiation potentials [2]. In our previous study [3], CD146-positive cells from human periodontal ligament were found to express MSC surface markers (CD105, CD90, CD73, CD44, and Stro-1) and show higher proliferative and osteogenic potential than CD146-negative cells. In addition, PDLSCs are also capable of regenerating periodontal tissues, including periodontal ligaments, alveolar bone, cementum, and peripheral nerves and blood vessels in animal models [4]. Clinical case reports [5] also show that autologous periodontal ligament progenitor cell transplantation in intrabony defect of 3 periodontitis patients achieves significant alveolar bone regeneration as well as attachment gain without adverse effects, indicating that PDLSC transplantation is a promising approach for regenerating periodontal tissues.

TNF-*α*, one of the most critical inflammatory factors in the progress of periodontitis [6], is mainly produced by activated macrophages, natural killer cells, T lymphocytes, and B lymphocytes. It is reported that there is a positive correlation between the concentration of TNF-*α* in the gingival crevicular fluid and periodontal inflammatory status [7]. TNF-*α* regulates cell proliferation, differentiation, and apoptosis by binding to its membrane-bound receptors [8]. TNFR1, a 55 kDa membrane protein containing a death domain on its intracellular region, is expressed in almost all cell types. TNFR1 participates in the regulation of cell proliferation, apoptosis, and differentiation through activation of NF-*κ*B, AP-1, or caspase-8. In contrast, TNFR2 does not have a death domain on its intracellular region and is expressed in several types of cells, including astrocytes, myocytes, thymocytes, endothelial cells, and human mesenchymal stem cells. Despite lacking the death domain, TNFR2 also regulates cell proliferation and differentiation through activation of NF-*κ*B and AP-1. Besides, TNFR2 regulates the rate of combination between TNF-*α* and TNFR1, possibly by increasing the local concentration of TNF-*α* at the cell surface through rapid ligand passing mechanism [9]. In our previous study [3], CD146-positive PDLSCs were more sensitive to TNF-*α* treatment in terms of proliferation inhibition when compared with CD146-negative periodontal fibroblasts. We also found that protein expression of both TNFR1 and TNFR2 in CD146-positive PDLSCs was 2-fold higher than that of CD146-negative periodontal ligament cells. However, which type of TNF receptor is mainly responsible for the effects of TNF-*α* in PDLSCs remains unclear.

It is well established that diabetes mellitus increases the risk and severity of periodontitis, especially in patients with poor metabolic control [10]. Indeed, periodontitis is considered the sixth complication of diabetes. Hyperglycemia, the most typical symptom of diabetes, has detrimental effects on cell proliferation, differentiation, and even causes cell death, leading to periodontal wound-healing delay. It is reported that high glucose inhibits proliferation and induces caspase-3-dependent apoptosis in periodontal ligament fibroblasts [11]. High glucose also hinders proliferation and osteogenic differentiation of PDLSCs by increasing the intracellular ROS level [12].

It has been reported that the average level of TNF-*α* in the gingival crevicular fluid (GCF) of periodontitis patients increases up to 9.5 ng/ml [13]. In addition, diabetic status upregulates monocytic TNF-*α* secretion (4.6-fold increase) of periodontitis patients in the presence of Gram-negative bacterial challenge [14]. In ligature-induced periodontitis animal study, the expression of TNF-*α* in periodontal tissues is also higher in Goto-Kakizaki rats (type 2 diabetes model) than that of the normoglycemic Wistar rats [15]. These data suggest that the diabetic condition may aggravate the detrimental effects of TNF-*α* in the periodontium. Hyperglycemia and a high concentration of TNF-*α* are two common pathological conditions in diabetic periodontitis patients, but how they affect PDLSCs is still unknown. Therefore, the aim of this study was to investigate how high glucose and TNF-*α* affect cell proliferation and apoptosis in human PDLSCs and to clarify possible mechanisms involved in this process. For this, human periodontal ligament stem cells were incubated with TNF-*α* under high-glucose conditions, and cell proliferation and apoptosis were detected. The function of two types of TNF receptors was also investigated. The intracellular ROS expression was examined, and the protective effect of vitamin C was also evaluated.

## 2. Materials and Methods

### 2.1. Cell Culture

The primary culture of periodontal ligament cells (PDLCs) was performed as described in our previous study [3]. Briefly, premolars or third molars were collected from twenty healthy persons aged from 12 to 24 years old for orthodontic reasons in the hospital of stomatology, Sun Yat-sen University. Periodontal ligament tissues were gently separated from the middle part of the root surface. Periodontal ligament tissues were cut into small pieces (1 mm^3^) followed by digestion with 2 mg/ml type 1 collagenase (Sigma-Aldrich, St. Louis, MO, USA) for 30 min at 37°C. After being passed through a 70 *μ*m strainer, cells were collected and suspended in *α*-MEM (12571071, Gibco/Thermo Fisher, USA) containing 10% (*v*/*v*) FBS, 100 U/ml penicillin, and 100 mg/ml streptomycin (all from Gibco, USA). All cells were incubated in a humidified atmosphere of 5% CO_2_ and 95% air at 37°C. The culture medium was changed every three days. The cells were subcultured when they reached 70% confluence using 0.25% (*w*/*v*) trypsin and 0.02% (*w*/*v*) EDTA (trypsin/EDTA; Gibco, USA).

### 2.2. Isolation and Identification of Human Periodontal Ligament Stem Cells

Human periodontal ligament stem cells were isolated using human CD146 microbead kit with LS columns and a MidiMACS separator (Miltenyi Biotec, Bergisch Gladbach, Germany) according to product instruction. In brief, passage-3 PDLCs (1 × 10^7^ cells) were prepared as single-cell suspensions using trypsin/EDTA buffer and resuspended in 60 *μ*l D-PBS buffer containing 0.5% bovine serum albumin and 2 mM EDTA. 20 *μ*l of FcR blocking reagent and 20 *μ*l of CD146 microbeads were added. The mixture was then incubated at 4-8°C for 15 min. Then, the cells were washed with 1 ml D-PBS buffer, centrifuged at 300 × g for 10 min, and resuspended in 500 ml D-PBS buffer. Magnetic separation was performed by applying the cell suspensions to an LS column, which was placed in the magnetic field of a MidiMACS separator. The column was washed with buffer three times so that unlabeled cells were washed away. The CD146 labeled cells were collected by removing the column from the magnetic field and flushing the column with 5 ml D-PBS buffer. CD146-positive cells were centrifuged at 300 × g for 10 min and resuspended in a culture medium.

For analysis of mesenchymal stem cell surface markers, cells from the second passage of CD146+PDLCs after magnetic bead isolation were analyzed using a Human MSC Analysis Kit (BD Stemflow, BD Biosciences, San Diego, CA, USA) for CD105, CD90, CD73, CD44, CD34, CD11b, CD19, CD45, and HLA-DR according to the product manual. Briefly, CD146+PDLCs were suspended in a buffer solution at 5 × 10^6^/ml density. Antibodies (PE-mouse anti-human CD44, FITC-mouse anti-human CD105, FITC-mouse anti-human CD90, APC-mouse anti-human CD73, hMSC positive/negative isotype control cocktail, or hMSC positive/negative cocktail) were added into 100 *μ*l cell samples, respectively. Samples were incubated at 4°C for 30 min in the dark and analyzed by flow cytometer (FACSCalibur, BD Biosciences, USA).

For the colony-forming assay, the first passage of CD146+PDLCs was seeded (150 cells per dish) in 10 cm-diameter culture dishes (Corning, NY, USA). After 14 days of culture, cells were fixed in 4% paraformaldehyde (Sigma-Aldrich, St. Louis, MO, USA) and stained with 0.1% toluidine blue (Sigma-Aldrich, St. Louis, MO, USA) for 5 min. The number of colonies per dish was determined visually under a light microscope (Axiovert 40, Carl Zeiss, Germany). Colonies containing more than 50 cells were considered one colony.

### 2.3. Immunohistochemical Staining of Stro-1, Cytokeratin, and Vimentin for CD146+PDLCs

CD146+PDLCs were cultured for 14 days, fixed in 4% paraformaldehyde, and blocked with 10% goat serum (Bosterbio, USA). The cells were incubated with mouse anti-human Stro-1 antibody (1 : 100), mouse anti-cytokeratin antibody (1 : 200), or mouse anti-Vimentin antibody (Clone V9,1 : 200) (all from Invitrogen Life Technologies, Carlsbad, CA, USA) for 2 h at 37°C. The samples were then incubated with rabbit anti-mouse immunoglobulin (IgG H + L, Miaotong Biology, China) for 1 h at 37°C and stained with 3,3′-diaminobenzidine (DAB) for 3 min and hematoxylin for 1 min. Samples were observed under a microscope (Axio Scope, Zeiss, Germany).

### 2.4. Osteogenic and Adipogenic Differentiation of CD146+PDLCs

CD146+PDLCs were seeded in 6-well dishes (5 × 10^4^ cells per well) and cultured for 2 days. Then, cells were cultured with osteogenic medium (containing 10% FBS, 0.1 *μ*M dexamethasone, 0.2 mM ascorbic acid, and 10 mM *β*-glycerophosphate; all from Sigma-Aldrich, St. Louis, MO, USA) or adipogenic medium (10%FBS, 1 *μ*M dexamethasone, 200 *μ*M indomethacin, and 0.5 mM 3-isobutyl-1-methylxanthine; all from Sigma-Aldrich, St. Louis, MO, USA) for 21 days or 14 days, respectively. The osteogenic and adipogenic medium were changed every 3 days. Cells were fixed in 4% paraformaldehyde and stained with 1% alizarin red for osteogenesis assay or 3 mg/ml oil red O for adipogenesis analysis (both from Sigma-Aldrich, St. Louis, MO, USA). Photographs were captured using an inverted microscope (Zeiss Germany, Oberkochen, Germany).

### 2.5. High-Glucose and TNF-*α* Treatment

Periodontal ligament stem cells were cultured under several different conditions as follows:

1. Control group (G5.6 group): cells were incubated in a medium containing 5.6 mM D-glucose (Sigma-Aldrich, St. Louis, MO, USA)
2. TNF-*α* treatment group (G5.6+TNF-*α* group): cells were incubated in a medium containing 5.6 mM D-glucose and TNF-*α* (10 ng/ml, Sigma-Aldrich, St. Louis, MO, USA);
3. High-glucose treatment group (G30 group): cells were incubated in a medium containing 30 mM D-glucose
4. High-glucose and TNF-*α* treatment group (G30+TNF-*α* group): cells were incubated in a medium containing 30 mM D-glucose and TNF-*α* (10 ng/ml)

The osmotic effect was also evaluated by the addition of mannitol (Sigma-Aldrich, St. Louis, MO, USA). Cells were incubated in a medium containing 5.6 mM D-glucose and 24.4 mM mannitol.

### 2.6. Tumor Necrosis Factor Receptor Blockage and Vitamin C Treatment

For TNFR1 or TNFR2 blockage experiments, mouse anti-human TNFR1 monoclonal antibody (10 *μ*g/ml, MAB625, R&D Systems, Minneapolis, MN, USA) or mouse anti-human TNFR2 monoclonal antibody (10 *μ*g/ml, MAB726, R&D Systems, Minneapolis, MN, USA) was added into the medium 1 hour before high-glucose and TNF-*α* treatment. Mouse IgG1 antibody was used as an isotype control (10 *μ*g/ml, MAB002, R&D Systems, Minneapolis, MN, USA). One hour later, the medium was replaced by a medium containing D-gl ucose (30 mM) and TNF-*α* (10 ng/ml). Then, another freshly prepared antibody was simultaneously added into the medium at a final concentration of 10 *μ*g/ml. The medium was changed every 3 days following the same protocol.

With regard to vitamin C treatment, ascorbic acid (200 *μ*M, A4544, Sigma-Aldrich, St. Louis, MO, USA) was added into medium free of ascorbic acid (A1049001, Gibco/Thermo Fisher, USA) 1 hour before high-glucose and TNF-*α* treatment. One hour later, the medium was replaced by a medium containing D-glucose (30 mM) and TNF-*α* (10 ng/ml). And another freshly prepared vitamin C solution was simultaneously added into the medium at a final concentration of 200 *μ*M. The medium was changed every 3 days following the same protocol.

### 2.7. Cell Viability Assay and Growth Curve of PDLSCs

Cell viability was evaluated by CCK-8 kit (Dojindo, Japan). PDLSCs were seeded in 96-well plates (2 × 10^3^ cells per well) and cultured for 6 days under different conditions as planned. Cell viability was tested every 24 hours: relative cell viability (%) = (OD_TNF−*α*_–OD_background_)/(OD_control_–OD_background_) × 100%. Growth curves were drawn by SPSS software version 22.0 (IBM, Armonk, NY, USA) using the *Y* = *A* × *e*^*B*×time^ model.

### 2.8. Western Blotting

Briefly, total proteins were extracted from the cells using RIPA lysis buffer (1% Triton X-100, 150 mM NaCl, 50 mM Tris, pH 7.4) and protease inhibitor mix, (all from Beyotime Institute of Biotechnology, Jiangsu, China) and an ultrasonic cell disruptor (Branson Instrument Co., Danbury, CT, USA). The protein concentrations were determined by BCA assay (Beyotime Institute of Biotechnology, Jiangsu, China). Equal protein samples (30-40 *μ*g) were separated on 10% SDS-PAGE gels (Beyotime Institute of Biotechnology, Jiangsu, China) and transferred onto the polyvinylidene difluoride membranes (Millipore, USA). Membranes were incubated with primary antibodies: rabbit anti-human TNFR1 (1 : 1000), rabbit anti-human TNFR2 (1 : 10 000; both from Epitomics, Abcam, Burlingame, CA, USA), and mouse anti-human *β*-actin (1 : 1000, Boster, Wuhan, China). Secondary antibodies were goat anti-mouse IgG-HRP (1 : 1000) and goat anti-rabbit IgG-HRP (1 : 1000; both from Boster, Wuhan, China). Images were obtained by chemiluminescence using an ECL-Western blot kit (CW Biotech, Beijing, China) and analyzed using ImageJ software (1.44p, USA).

### 2.9. Cell Cycle Analysis

PDLSCs were plated in 25 cm^2^ flask (1.67 × 10^4^ cell per flask, Corning, NY, USA) and cultured for 24 hours. Then, cells were incubated under different conditions as planned for 4 days. Cells were harvested and fixed in 70% ethanol at 4°Cfor 24 h. Cells were washed twice with ice-cold phosphate buffer saline (PBS, Gibco, U.S.) and incubated with RNase and propidium iodide (F10797, Thermo Fisher, Waltham, MA, USA) for 30 min, and then cell cycle analysis was performed by a flow cytometer (FACSCalibur, BD Biosciences, USA). Relative S phase fraction (%) was calculated from dividing the S phase fraction of the test group by that of the control group.

Cell proliferation was detected by 5-ethynyl-2′-deoxyuridine (EdU) incorporation assay following the product instruction (C10632, Thermo Fisher, Waltham, MA, USA). In brief, PDLSCs were plated in 25 cm^2^ flask (1.67 × 10^4^ cell per flask, Corning, NY, USA) and cultured for 24 hours. Then, cells were incubated under different conditions as planned for 3 days. EdU reagent was added into the culture medium at a final concentration of 10 *μ*M. 24 hours later, cells were harvested, fixed, and permeabilized in the tube. Cells were incubated with Click-iT™ Plus reaction cocktails for 30 min at room temperature in the dark. Cells were washed once with 3 ml of wash reagent and analyzed by a flow cytometer (FACSCalibur, BD Biosciences, USA).

### 2.10. Detection of Intracellular Reactive Oxygen Species

PDLSCs were plated in 25 cm^2^ flask (1.67 × 10^4^ cell per flask, Corning, NY, USA) and cultured for 24 hours. Then, cells were incubated under different conditions as planned for 6 days. Cells were harvested and resuspended in 1 ml culture medium containing cellROX reagent (final concentration 0.02%, Thermo Fisher, Waltham, MA, USA). Cells were incubated at 37°C for 60 min, and then intracellular ROS expression was detected by a flow cytometer (FACSCalibur, BD Biosciences, USA). The relative intracellular ROS level was calculated from dividing the ROS level of the test group by that of the control group.

### 2.11. Detection of Cell Apoptosis

Cell apoptosis on day 2 was detected using TUNEL (TdT-mediated dUTP nick-end labeling) assay kit (Roche, Mannheim, Germany). In brief, PDLSCs were plated in 48-well plates (1.0 × 10^4^ cell per well) and cultured for 24 hours. Then, cells were incubated under different conditions as planned for 2 days. Cells were fixed in 4% paraformaldehyde for 1 hour, incubated in 3% H_2_O_2_ for 10 min, and permeabilized in the reagent containing 0.1% trisodium citrate and 0.1% Triton X-100 for 2 min on ice. Then, cells were labeled with fluorescein-dUTP and peroxidase-labeled anti-fluorescein antibody. The TUNEL reaction was visualized with diaminobenzidine (DAB). A dark accumulation of DAB in the cells (nuclei and apoptotic bodies) was judged to indicate a positive reaction to TUNEL. The nuclei of cells were stained with 500 ng/ml DAPI (Roche, Mannheim, Germany). For each well, at least 10 random microscope fields (100x) were examined using an inverted fluorescence microscopy (Zeiss Germany, Oberkochen, Germany) and analyzed using Image-Pro Plus software.

Cell apoptosis on day 6 was detected using Annexin-V/PI kit (Thermo Fisher, Waltham, MA, USA). PDLSCs were plated in 25 cm^2^ flask (1.67 × 10^4^ cell per flask, Corning, NY, USA) and cultured for 24 hours. Then, cells were incubated under different conditions as planned for 6 days. Cells were harvested by trypsin (EDTA free) and washed twice with PBS. Then, cells were incubated with Annexin-V and PI reagent in a binding buffer for 15 min in the dark. Cell apoptosis was analyzed by a flow cytometer (FACSCalibur, BD Biosciences, USA).

### 2.12. Statistical Analysis

All experiments were replicated 3 times using periodontal ligament stem cells obtained from 3 different individuals. A total of 20 healthy individuals were included in this study. Data were analyzed using SPSS software version 22.0 (IBM, Armonk, NY, USA). The data were expressed as mean ± standard deviation and analyzed using one-way ANOVA followed by post hoc LSD *t*-test. *p* values under 0.05 were considered statistically significant.

## 3. Results

### 3.1. Isolation and Identification of CD146-Positive Periodontal Ligament Stem Cells (PDLSCs)

After 7–14 days, primitive periodontal ligament cells (PDLCs) attached and started to proliferate. CD146-positive (CD146+) PDLCs were isolated from passage-3 PDLCs using immunomagnetic beads. Second passage CD146+PDLCs were positive for MSC markers, including CD105 (92.16%) (Fig. [Supplementary-material supplementary-material-1]), CD73 (99.11%), and CD90 (98.76%) (Fig. [Supplementary-material supplementary-material-1]). Besides, CD146+PDLCs were negative for hematopoietic markers CD34, CD11b, CD19, CD45, and HLA-DR (Fig. [Supplementary-material supplementary-material-1]).

First passage CD146+PDLCs were able to form colonies (Fig. [Supplementary-material supplementary-material-1]) after 14 days of culture at low cell density (150 cells per dish). Under a microscope, cells were found to be small, spindle shaped, and arranged in clusters. In addition, CD146+PDLCs were found to be positive for vimentin and negative for cytokeratin (Fig. [Supplementary-material supplementary-material-1], left and middle), which was consistent with stromal cell properties. With regard to Stro-1 (Fig. [Supplementary-material supplementary-material-1], right), another mesenchymal stem cell marker, some cells expressed Stro-1 (stained brown in the cytoplasm), whereas other cells were negative for Stro-1 (transparent cells). After osteogenic or adipogenic induction, CD146+PDLCs were found to form more calcium nodules (Fig. [Supplementary-material supplementary-material-1], left) and more lipid droplets (Fig. [Supplementary-material supplementary-material-1], right) than the CD146 PDLCs.

In summary, CD146-positive PDLCs were able to form colonies at low cell density, undergo osteogenesis and adipogenesis, express MSC surface markers CD90, CD105, CD73, and Stro-1 but not hematopoietic markers, indicating that CD146-positive cells from human periodontal ligament tissues were a subpopulation of mesenchymal stem cells. CD146-positive periodontal ligament cells (CD146+PDLCs) were considered periodontal ligament stem cells (PDLSCs) in the present study.

### 3.2. High Glucose Exacerbated TNF-*α*-Induced Cell Viability Inhibition in PDLSCs

**Figure 1:**
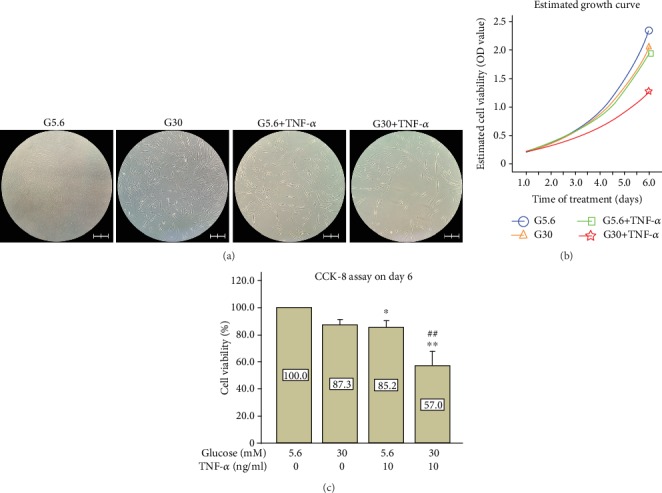
High glucose exacerbated the inhibitory effects of TNF-*α* on the cell viability of PDLSCs. PDLSCs were seeded on a 96-well plate and treated with 5.6 mM glucose (G5.6), 30 mM glucose (G30), 10 ng/ml TNF-*α* (G5.6+TNF-*α*), and 30 mM glucose combined with 10 ng/ml TNF-*α* (G30+TNF-*α*). (a) Morphology of PDLSCs on day 6 under a microscope. Scale bars represent 200 *μ*m. (b) Estimated growth curve for PDLSCs using the *Y* = *A* × *e*^*B*×*T*^ model. (c) CCK-8 assays on day 6. The cell viability of the control group was regarded as 100%. Data are expressed as means ± standard deviations. All assays were replicated 3 times using PDLSCs obtained from 3 different individuals. ^∗^*p* < 0.05 versus the control group (G5.6); ^∗∗^*p* < 0.01 versus the control group (G5.6); ^##^*p* < 0.01 versus the G5.6+TNF-*α* group.

CCK-8 assays were performed every 24 hours. Cell morphology on day 6 was recorded as well. After 6 days of culture, PDLSCs in the control group reached confluence (Figure 1(a), G5.6). The cell density of the high-glucose group (G30) was lower compared with the control group (Figure 1(a), G30), but the difference was not statistically significant (Figure 1(c)). However, TNF-*α* treatment inhibited PDLSC cell viability (85.2%, *p* < 0.05) compared with the control group (Figure 1(c)). There is more space between cells in the TNF-*α* group (Figure 1(a), G5.6+TNF-*α*) than in the control group. Interestingly, high glucose exacerbated the inhibitory effect of TNF-*α*. Cell density further decreased in the high-glucose and TNF-*α* treatment group (G30+TNF-*α*) compared with the TNF-*α* group (G5.6+TNF-*α* in Figure 1(a)). CCK-8 assays also showed similar results (Figure 1(c)). The estimated growth curve (Figure 1(b)) clearly revealed that G30+TNF-*α* treatment (Figure 1(b), red star) severely delayed the proliferation of PDLSCs compared with both the control group (Figure 1(b), blue circle) and the G5.6+ TNF-*α* group (Figure 1(b), green square). The osmotic effect of high glucose was evaluated by the addition of mannitol. Results (Fig. [Supplementary-material supplementary-material-1]) showed that mannitol treatment did not exacerbate TNF-*α*-induced proliferation inhibition on day 6, suggesting that the detrimental effect was caused by a high level of glucose rather than the change of osmotic pressure.

### 3.3. High Glucose Elevated Protein Expression of TNFR1 in PDLSCs

**Figure 2:**
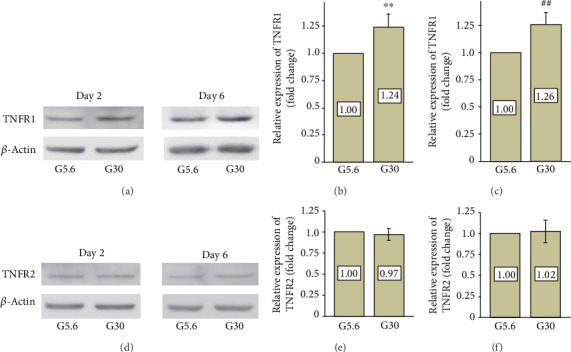
Protein expression of TNFR1 and TNFR2 in PDLSCs under high-glucose conditions. PDLSCs were cultured under normal glucose (G5.6) or high-glucose (G30) conditions. Protein expression of TNFR1 and TNFR2 was detected by western blotting. (a–c) Protein expression of TNFR1 was elevated by high-glucose treatment on day 2 and day 6. Data are expressed as means ± standard deviations. Experiments were replicated 3 times using PDLSCs obtained from 3 different individuals. ^∗∗^*p* < 0.01 versus the control group on day 2; ^##^*p* < 0.01 versus the control group on day 6. (d–f) Protein expression of TNFR2 was unchanged under high-glucose conditions on day 2 and day 6.

Since high glucose strengthened the inhibitory effects of TNF-*α* on cell viability in PDLSCs, we speculated that high glucose might increase the expression of TNF receptors. As shown in Figures 2(a)–2(c), high glucose (30 mM) elevated protein expression of TNFR1 in PDLSCs on day 2 (1.24-fold of the control group, *p* < 0.01) and day 6 (1.26-fold, *p* < 0.01). The protein expression of TNFR2 was also assessed (Figures 2(d)–2(f)), and results showed that TNFR2 expression was unchanged after exposure to high-glucose conditions for 2 or 6 days.

### 3.4. High Glucose and TNF-*α* Inhibited Cell Viability and Caused Cell Cycle Arrest by Activating TNFR1

In order to investigate which type of TNF receptor is responsible for high-glucose- and TNF-*α*-induced cell viability inhibition, specific antibodies targeting TNFR1 or TNFR2 were applied into the culture medium 1 hour before TNF-*α* treatment.

**Figure 3:**
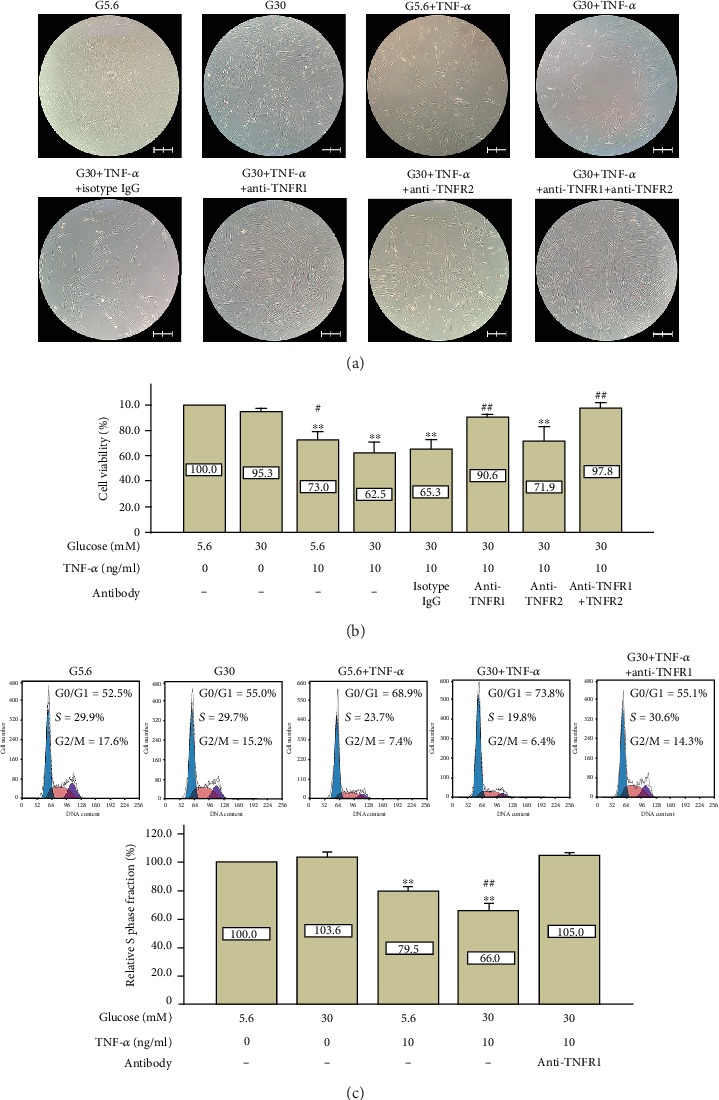
High glucose and TNF-*α* inhibited cell viability and caused cell cycle arrest by activating TNFR1. (a) Cell morphology after blockage of TNFRs by specific antibodies (day 6). Specific antibodies were used to block either TNFR1 or TNFR2. Cell morphology was observed under a microscope (100x). Scale bars represent 200 *μ*m. (b) CCK-8 assay on day 6. Specific antibodies were used to block either TNFR1 or TNFR2. The CCK-8 assay was performed to detect cell viability. Data are presented as means ± standard deviations. ^∗∗^*p* < 0.01 versus the control group (G5.6); ^#^*p* < 0.05 versus the G30+TNF-*α* group; ^##^*p* < 0.01 versus the G30+TNF-*α* group. (c) High glucose and TNF-*α* decreased the S phase proportion of PDLSCs (day 4). PDLSCs were cultured under different conditions for 4 days. The cell cycle was assessed by flow cytometry (PI staining). The relative S phase fraction of the control group was regarded as 100%. Data are presented as means ± standard deviations. ^∗∗^*p* < 0.01 versus the control group; ^##^*p* < 0.01 versus the G5.6+TNF-*α* group. All assays were replicated 3 times using PDLSCs obtained from 3 different individuals.

The blockage of both TNFR1 and TNFR2 totally reversed high-glucose- and TNF-*α*-induced cell viability inhibition (97.8% of the control group, *p* > 0.05). Cell density in the G30+TNF-*α*+anti-TNFR1+anti-TNFR2 group was recovered to the level of the control group (Figures 3(a) and 3(b)). Interestingly, the blockage of TNFR1 alone dramatically reversed high-glucose- and TNF-*α*-induced cell viability inhibition (90.6% of the control group, *p* > 0.05), even though it was lower than the control group, but there was no statistical significance. Cell density in the G30+TNF-*α*+anti-TNFR1 group was also recovered to the level of the control group (Figures 3(a) and 3(b)). However, the blockage of TNFR2 did show some recovery of cell density and cell viability under high-glucose and TNF-*α* conditions, but the difference is of no statistical significance (*p* > 0.05) (Figures 3(a) and 3(b)).

The cell cycle was also detected by flow cytometry on day 4. As shown in Figure 3(c), high glucose alone did not cause cell cycle arrest, whereas TNF-*α* treatment decreased the relative S phase fraction (79.5% of the control group, *p* < 0.01). Similar to the results of the CCK-8 assay, the relative S phase fraction of the G30+TNF-*α* group further decreased (66.0% of the control group, *p* < 0.01) compared with the G5.6+TNF-*α* group. Consistent with results of the CCK-8 assay, blockage of TNFR1 could totally restore the relative S phase fraction in the presence of high glucose and TNF-*α* (105% of the control group, *p* > 0.05).

The protein expression of cyclin-dependent kinase 4 (CDK4) was also evaluated by western blotting on day 6. As shown in [Supplementary-material supplementary-material-1], TNF-*α* treatment decreased CDK4 expression (39% of the control group, *p* < 0.01), and the expression of CDK4 in the G30+TNF-*α* group further decreased compared with the G5.6+TNF-*α* group (26% of the control group, *p* < 0.05).

**Figure 4:**
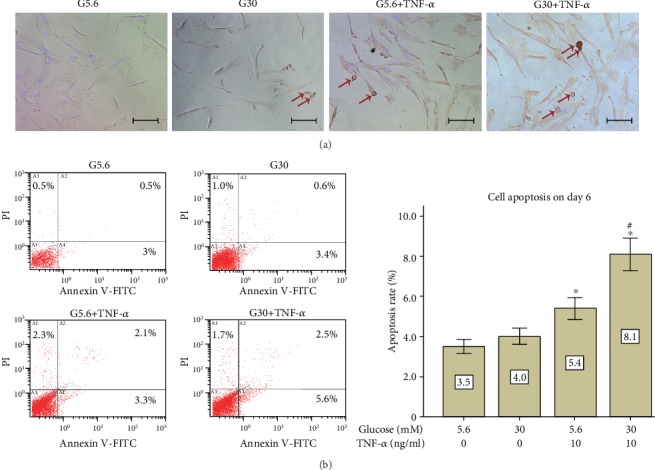
High glucose and TNF-*α* caused cell apoptosis in PDLSCs. PDLSCs were treated under different conditions (G5.6, G30, G5.6+TNF-*α*, and G30+TNF-*α*) for 2 days or 6 days. (a) TUNEL/DAPI double staining was used to detect apoptosis on day 2. Apoptosis cells were stained brown and indicated by red arrows. Scale bars represent 100 *μ*m. (b) Annexin V-FITC/PI double staining was conducted to detect apoptosis on day 6. The horizontal axis represents the fluorescent intensity of annexin V-FITC, whereas the vertical axis represents the fluorescent intensity of PI. Data are expressed as means ± standard deviations. All assays were replicated 3 times using PDLSCs obtained from 3 different individuals. ^∗^*p* < 0.05 versus the control group (G5.6), ^#^*p* < 0.05 versus the G5.6+TNF-*α* group.

**Figure 5:**
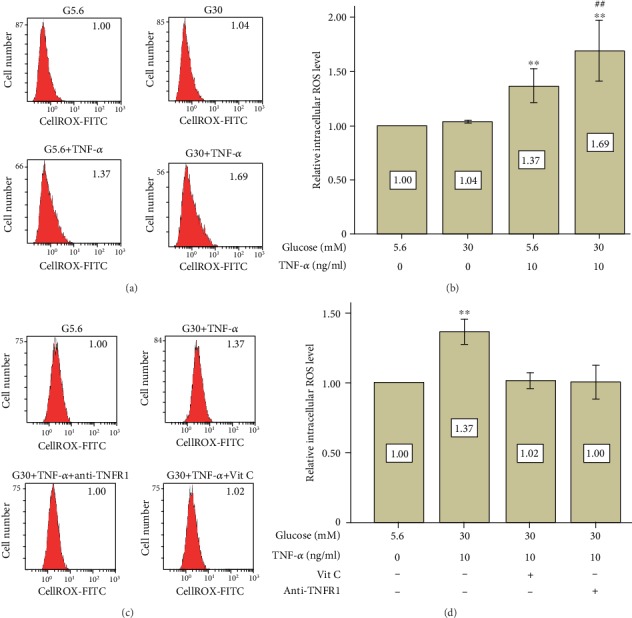
Addition of vitamin C or TNFR1 antibody totally reversed elevation of intracellular ROS expression caused by high glucose and TNF-*α*. PDLSCs were cultured under different conditions. Intracellular ROS level was detected by using CellROX regent and flow cytometry on day 6. (a) High glucose further increased TNF-*α*-induced intracellular ROS expression on day 6. Data are expressed as means ± standard deviations. All assays were replicated 3 times using PDLSCs obtained from 3 different individuals. ^∗∗^*p* < 0.01 versus the control group (G5.6), ^##^*p* < 0.01 versus the G5.6+TNF-*α* group. (b) Vitamin C or TNFR1 antibody reverses the elevation of intracellular ROS expression on day 6. Data are expressed as means ± standard deviations. All assays were replicated 3 times using PDLSCs obtained from 3 different individuals. ^∗∗^*p* < 0.01 versus the control group (G5.6).

In order to confirm if the decline in cell viability was due to proliferative inhibition rather than cell apoptosis, which is quite a common phenomenon caused by TNF-*α* treatment, cell apoptosis was evaluated by both TUNEL/DAPI staining (Figure 4(a)) and Annexin V/PI staining (Figure 4(b)). Results show that even though the apoptosis rate was higher in the G30+TNF-*α* group compared with the control group (8.1% vs. 3.5%, *p* < 0.01), the increase of apoptosis cells is relatively small (4.6%). In contrast, the change of the relative S phase fraction in the G30+TNF-*α* group was intense (66.0% of the control group, Figure 3(c)), so did the change of cell viability in the G30+TNF-*α* group (62.5% of the control group, Figure 5(b)). These results indicate that the decrease in cell viability and cell density was mainly caused by the inhibition of cell proliferation rather than cell apoptosis.

### 3.5. Addition of Vitamin C or TNFR1 Antibody Totally Reverses Elevation of Intracellular ROS Expression Caused by High Glucose and TNF-*α*

It is reported that both high glucose and TNF-*α* can upregulate intracellular ROS expression in many cell types. Therefore, the intracellular ROS level was investigated by using CellROX regent and flow cytometry on day 6. As expected, TNF-*α* treatment increased ROS expression (1.37-fold, *p* < 0.01), and high glucose further promoted the elevation of intracellular ROS in the presence of TNF-*α* (1.69-fold, *p* < 0.01) (Figures 5(a) and 5(b)). The antioxidant vitamin C (200 *μ*M) was found to be able to reverse the elevation of ROS expression caused by high glucose and TNF-*α* (1.02-fold of the control group, *p* > 0.05) (Figures 5(c) and 5(d)). Similarly, blockage of TNFR1 also prevented ROS elevation in the G30+TNF-*α* group (1.00-fold of the control group, *p* > 0.05) (Figures 5(c) and 5(d)), suggesting that ROS expression is caused by activation of TNFR1.

### 3.6. Vitamin C Partially Restored Cell Proliferation in the Presence of High Glucose and TNF-*α*

**Figure 6:**
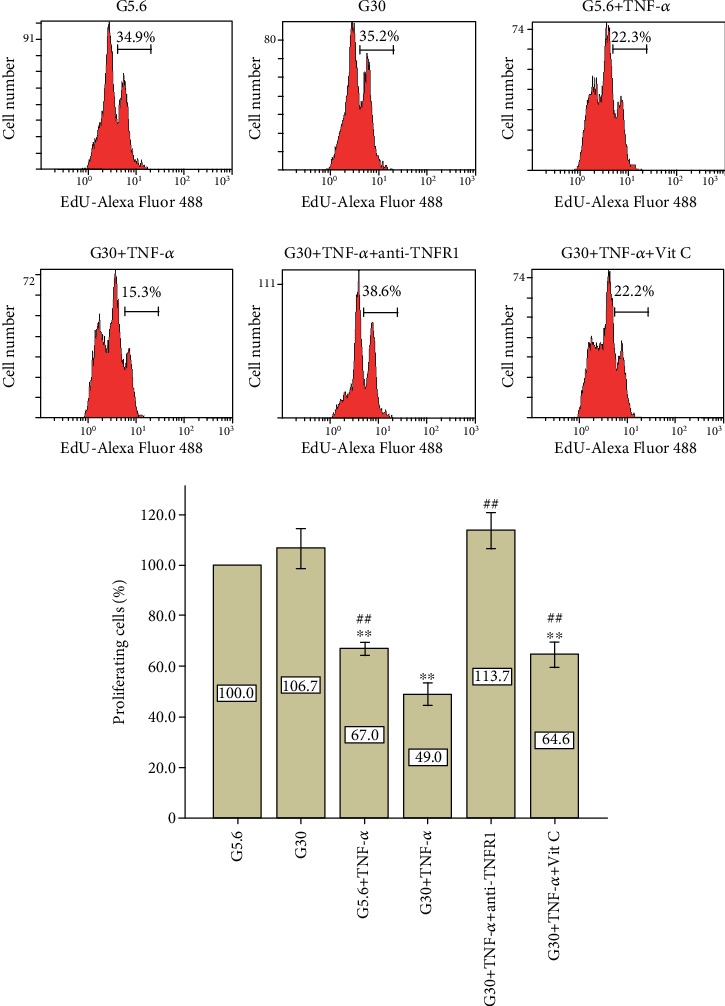
Vitamin C partially restored cell proliferation in the presence of high glucose and TNF-*α*. PDLSCs were cultured under different conditions. Cell proliferation was detected by EdU assay on day 4. Data are presented as means ± standard deviations. This assay was replicated 3 times using PDLSCs obtained from 3 different individuals. ^∗∗^*p* < 0.01 versus the control group; ^##^*p* < 0.01 versus the G30+TNF-*α* group.

EdU incorporation assay was performed to verify whether the blockage of ROS can reverse proliferative inhibition caused by high glucose and TNF-*α*.  Figure 6 shows that proliferating cells in the G30+TNF-*α* group (49.0%, *p* < 0.01) were severely decreased compared with the control group (100.0%) and G5.6+TNF-*α* group (67.0%) (Figure 6). However, the addition of vitamin C partially restored cell proliferation (64.6%) in the presence of high glucose and TNF-*α*. The blockage of TNFR1 by specific antibody could totally reverse the inhibitory effect of high glucose and TNF-*α* treatment in terms of cell proliferation (113.7%).

**Figure 7:**
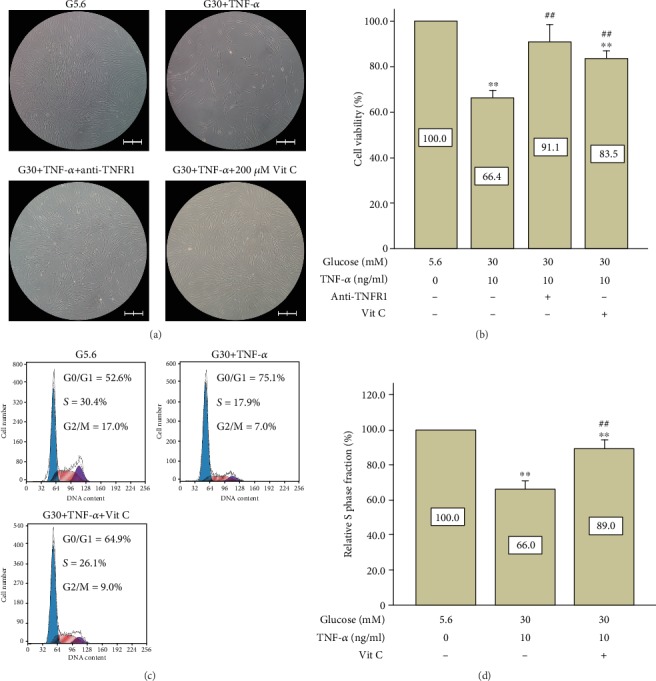
Administration of vitamin C partially restored cell viability and S phase fraction in PDLSCs treated with high glucose and TNF-*α*. (a) Cell morphology was observed under a microscope (100x, scale bars represents 200 *μ*m). (b) Vitamin C partially restored cell viability on day 6 (CCK-8 assay). Data are expressed as means ± standard deviations. All assays were replicated 3 times using PDLSCs obtained from 3 different individuals. ^∗^*p* < 0.05 versus the control group (G5.6), ^#^*p* < 0.05 versus the G30+TNF-*α* group. (c) Vitamin C partially restored the S phase fraction on day 4 (cell cycle analysis by flow cytometry). The relative S phase fraction of the control group was regarded as 100%. Data are presented as means ± standard deviations. All assays were replicated 3 times using PDLSCs obtained from 3 different individuals. ^∗∗^*p* < 0.01 versus the control group; ^##^*p* < 0.01 versus the G30+TNF-*α* group.

Similar results were found in both CCK-8 assay and cell cycle detection. Cell density was considerably restored in the vitamin C treatment group in the presence of high glucose and TNF-*α* (Figure 7(a)). Similarly, cell viability revealed by CCK-8 assay in the vitamin C treatment group was also recovered to a large extent (83.5% of the control group, Figure 7(b)). As expected, vitamin C treatment also recovers relative S phase fraction from 66.0% to 89.0% in the presence of high glucose and TNF-*α* (Figures 7(c) and 7(d)).

**Figure 8:**
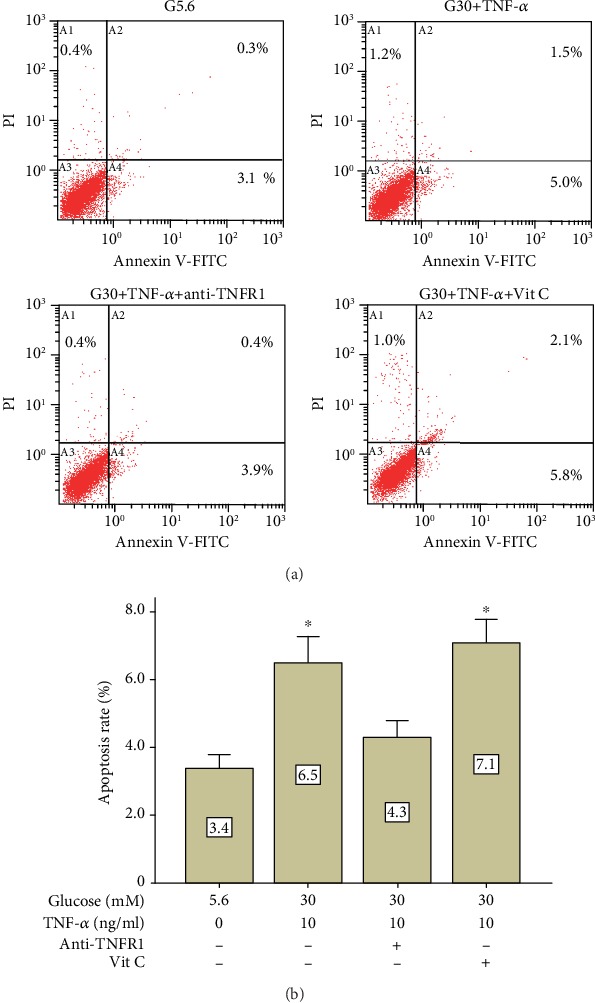
Vitamin C did not alter cell apoptosis in PDLSCs treated with high glucose and TNF-*α*. Flow cytometry detection of apoptosis by annexin V-FITC/PI double staining (day 6). The horizontal axis represents the fluorescent intensity of annexin V-FITC, whereas the vertical axis represents the fluorescent intensity of PI. Data are expressed as means ± standard deviations. All assays were replicated 3 times using PDLSCs obtained from 3 different individuals. ^∗^*p* < 0.05 versus the control group (G5.6).

In order to confirm whether the beneficial effect of vitamin C on cell viability is caused by alleviating apoptosis or not, annexin V/PI staining assay was performed. Results showed that (Figure 8) cell apoptosis induced by high-glucose and TNF-*α* treatment was not altered by the addition of vitamin C, supporting that the decrease in cell viability mainly resulted from proliferative inhibition rather than cell apoptosis caused by high-glucose and TNF-*α* treatment.

## 4. Discussion

Stem cells in periodontal tissues are considered a group of heterogeneous cells. To date, there is no single specific biomarker for isolation and identification of stem cells in periodontal ligament [16]. It is reported that STRO-1, SSEA-4, CD271, and CD146 are used as indicators for the enrichment of periodontal ligament cells [17] that possess the ability of self-renewal and multiple differentiation potential. Chen and colleagues' study [18] showed that localization of putative MSCs in healthy human teeth and the periodontitis affected teeth are near the blood vessels. In addition, it is now believed that MSCs also originate from pericytes [17], a cell population that localized near blood vessels and is responsible for blood vessel nutrition and stabilization. Pericytes possess mineralization activity [19], as well as multipotency [20], share some similarities with PDLSCs including the expression of NG2, CD146, and CD140b, and participate in the maintenance of blood vessel-like structures [21]. Therefore, in the present study, CD146 was used as a biomarker for the identification of the potent progenitor cells in the periodontal ligament. Furthermore, it is inevitable that stem cells lose their differentiation potentials and self-renewal ability under in vitro conditions [22], no matter which kind of biomarker is used for purification. In the present study, CD146-positive PDLCs within passage 5 were used in order to minimize the detrimental effect of in vitro culture conditions.

Impaired periodontal wound healing is a common symptom of diabetic periodontitis patients. It is reported that the blood glucose levels of Akita mice (type 1 diabetes animal model) reach 600 mg/dl (33.3 mM) at the age of 3 weeks [23]. Glucose at 30 mM was used as high-glucose conditions in in vitro studies [24, 25]. It is reported that high glucose impairs cell survival by inhibiting proliferation and inducing cell apoptosis. Cell viability of fibroblasts in the periodontal ligament is inhibited (87.1% of the control group, *p* < 0.05) under high-glucose conditions (25 mM) for 7 days [26]. In addition, periodontal ligament cell apoptosis rate is also upregulated from 3% to 5% when cells are incubated in 25 mM high-glucose medium for 24 hours [11]. However, in the present study, cell proliferation of PDLSCs was unchanged under high-glucose condition (30 mM) for 6 days, so was cell apoptosis. These results indicate that PDLSCs are more resistant to high glucose than periodontal fibroblasts.

According to clinical researches, there is a positive correlation between the severity of periodontal inflammation and the level of TNF-*α* in gingival crevicular fluids (GCF). The concentration of TNF-*α* in GCF of periodontally healthy individuals is 2.73 ng/ml [13], and it increases up to 9.5 ng/ml in experimental gingivitis patients [27] and chronic periodontitis patients [28]. In contrast to high glucose, proliferation and apoptosis of PDLSCs are affected by TNF-*α* treatment in a dose-dependent manner. In our previous study [3], CD146-positive PDLSCs were treated with TNF-*α* at different concentrations for 48 hours. TNF-*α* at 2.5 ng/ml and 5 ng/ml were found to promote cell proliferation, but TNF-*α* at 10 ng/ml showed the apoptotic effect on PDLSCs, suggesting that severe inflammatory condition is detrimental to PDLSCs. As expected, in the present study, PDLSCs incubated with TNF-*α* at 10 ng/ml for 6 days showed lower cell viability and higher apoptosis rate compared with the control group. However, the apoptosis rate of the G30+TNF-*α* group was only 4% higher than that of the control group, suggesting that the decrease in cell viability was mainly caused by proliferative inhibition.

In this study, the harmful effects of TNF-*α* were significantly amplified in the presence of high glucose even though high glucose itself did not do harm to PDLSCs. Esposito and colleagues revealed that TNF-*α* concentration was acutely increased by hyperglycemia in human subjects through an oxidative mechanism [29], and the excessive TNF-*α* is closely related to diabetic complications by inducing endothelial cell apoptosis under high-glucose condition [30]. Since the effect of TNF-*α* is based on its concentration, we speculated whether high glucose exacerbated inhibitory effects of TNF-*α* by increasing its expression. After culture in a high-glucose medium (30 mM) with or without TNF-*α* treatment for 2 days, the level of TNF-*α* in the cell supernatant and cell lysate was measured by enzyme-linked immunosorbent assay (Fig. [Supplementary-material supplementary-material-1]). Results showed that TNF-*α* in the cell supernatant was below the threshold value (5.6 pg/ml), which is consistent with Shu and colleagues' finding that basal expression of TNF-*α* in periodontal ligament cells is about 10 pg per 10^6^ cells. On the contrary, the concentration of TNF-*α* in the cell lysate of the G30+TNF-*α* group was 30% higher than that of the G5.6+TNF-*α* group. These results indicate that high glucose facilitated the binding between exogenous TNF-*α* and TNF receptors rather than increased the endogenous expression of TNF-*α*.

Furthermore, the expression of two kinds of TNF receptors was detected by western blotting. Protein expression of TNFR1 was increased by high glucose, whereas expression of TNFR2 was unchanged under high-glucose conditions. The expression of TNFR1 can be upregulated by several transcription factors, including CCAAT/enhancer binding protein (C/EBP), NF-*κ*B, and activating protein 1 (AP-1) [31]. C/EBP, NF-*κ*B, and AP-1 are found to be activated in hyperglycemia conditions and are involved in the development of diabetic complications [32, 33]. These transcription factors are also activated by TNF-*α*, suggesting a positive feedback regulation. However, further research is needed to clarify whether the upregulation of TNFR1 in high-glucose conditions is mediated by C/EBP, NF-*κ*B, or AP-1. The functions of TNFR1 and TNFR2 under high-glucose conditions were also evaluated using specific antibodies. TNFR1, instead of TNFR2, was found to be responsible for proliferative inhibition and apoptosis. Our results are in accordance with other researchers' findings that hepatocyte apoptosis [34] and retinal neural cell apoptosis [35] caused by diabetic hyperglycemia are mediated by the activation of TNFR1. In the present study, blockage of TNFR2 partly restored cell viability (even though the difference was not statistically significant), which could be due to the affinity between TNF-*α* and TNFR2 being far lower than that between TNF-*α* and TNFR1. Therefore, TNFR2 acts as a ligand-passing machine that facilitates the function of TNFR1 [36].

Clinical researches and animal studies reveal that total antioxidant capacities of GCF are inversely proportional to the extent of periodontal inflammation. Pavlica and colleagues [37] using a canine model discovered that total antioxidant capacities of GCF in the mild periodontitis group are significantly higher than that in the severe periodontitis group. Moreover, Brock and colleagues [38] found similar results in periodontitis patients, suggesting that oxidative stress may be involved in the progression of periodontitis [39]. On the one hand, the overproduction of reactive oxygen species (ROS) by the mitochondrial electron transport chain is considered to be an essential pathogenic mechanism in the process of diabetic complications. Under the hyperglycemia condition, the excessive glucose-derived pyruvate is oxidized in the tricarboxylic acid cycle, which increases the flux of NADH and FADH_2_ into the electron transport chain. The overloaded electron transport chain generates ROS by passing electrons to molecular oxygen [40]. On the other hand, ROS production can also be triggered by TNF-*α*. It is reported that human skin fibroblasts release ROS in a time- and dose-dependent manner after stimulation with TNF-*α* [41]. What is more, TNF-*α*-induced ROS elevation in vascular smooth muscle cells is further augmented in high-glucose conditions [42], indicating a synergistic effect between TNF-*α* and high glucose. It is reported that the addition of exogenous ROS or agents that trigger an increase in endogenous ROS generation sensitize many types of cells (endothelial cells, hepatocytes, and fibrosarcoma cells) to TNF-induced apoptosis possibly through inhibition of NF-*κ*B transcription of survival genes [43]. In the present study, TNF-*α*-induced ROS expression was also further increased in the presence of high glucose. However, PDLSC apoptosis was not alleviated by the treatment of vitamin C, suggesting that other mechanisms are involved in this process. In contrast, the decrease in cell viability caused by high-glucose and TNF-*α* treatment was markedly reversed by vitamin C treatment, indicating that ROS plays a crucial role in cell cycle arrest. It is well established that DNA damage caused by ROS can activate p53, which in turn upregulates p21^cip1^. The upregulation of p21^cip1^ inhibits the activation of CDK2 and induces cell cycle arrest in the G1 phase [44]. Also, expression of p16^INK4a^ is also upregulated by elevated ROS, which binds to CDK4/6 and arrests cells in the G1 phase [44]. In the present study, the cell cycle analysis (Figure 3(c)) revealed that PDLSCs incubated with TNF-*α* under high-glucose condition exhibited cell cycle arrest in the G0/G1 phase (73.8%) compared with the control group (52.5%). Blocking ROS production by 200 *μ*M vitamin C markedly reduced the proportion of G0/G1 phase from 75.1% to 64.9% (Figure 7(c)) and restored S phase fraction from 17.9% to 26.1% in the presence of high glucose and TNF-*α*, suggesting an essential role of ROS in the regulation of cell cycle in PDLSCs. Further researches are needed to verify whether p21^cip1^ or p16^INK4a^ is involved in this process.

Vitamin C (ascorbic acid) is a water-soluble antioxidant that can scavenge several types of oxygen radicals, including singlet oxygen and hydroxyl radicals. The concentration of vitamin C in GCF is 207.3 *μ*M, which is 3-fold higher than plasma concentration (72 *μ*M) [45]. In addition, the plasma level of vitamin C in chronic periodontitis patients is 50% lower than periodontal healthy individuals [46]. Therefore, in this study, vitamin C at 200 *μ*M was used as an antioxidant and exhibited excellent protective effects on PDLSCs in terms of cell proliferation. It is reported by Wei and colleagues [47] that vitamin C at 20 *μ*g/ml (113.6 *μ*M) is capable of enhancing the proliferation capacity and osteogenic differentiation efficiency of PDLSCs through inducing telomerase activity. However, the administration of vitamin C only partially reverse TNF-*α*- and high glucose-induced cell cycle arrest in PDLSCs, suggesting that there are other signaling pathways other than ROS involved in TNF-*α*-mediated cell cycle arrest. For example, it is reported that p53 activation, as well as p21 accumulation, is not necessary for TNF-*α*-induced G1 arrest. Instead, inhibition of Rb phosphorylation by TNF-*α* without activation of p53 is considered a possible mechanism [48]. In the present study, we also found that the expression of ERK1/2, as well as the expression of CDK4, was both reduced under high-glucose and TNF-*α* conditions (Figs. [Supplementary-material supplementary-material-1], [Supplementary-material supplementary-material-1], and [Supplementary-material supplementary-material-1]). It is well established that sustained activation of ERK1/2 is necessary for S-phase entry during cell proliferation [49]. Yan and colleagues [50] reported that vitamin C promoted proliferation and osteogenesis of PDLSCs by activation of ERK1/2. Therefore, further studies are needed to explore whether the beneficial effects of vitamin C depend on the activation of ERK1/2 under high-glucose and TNF-*α* conditions. Besides, we also tested the impact of liposoluble vitamin E (200 *μ*M) on cell proliferation ([Supplementary-material supplementary-material-1]). Vitamin E also partially restored cell viability on day 6 under high-glucose and TNF-*α* conditions, but the effect is weaker than that of vitamin C (*p* < 0.05). Frei and colleagues' study [51] revealed that oxygen radicals were preferentially reduced by vitamin C rather than vitamin E. The lipid peroxidation occurred only after plasma vitamin C depletion. If vitamin C were removed from the plasma, vitamin E could not completely block the occurrence of lipid peroxidation but only delay the rate of lipid peroxidation. These findings could explain why vitamin C exhibited better protective effects on PDLSCs when compared with vitamin E.

This study has potential limitations. First, PDLSCs were treated under a constant high-glucose condition (30 mM) for a relatively short time (6 days). Indeed, blood glucose fluctuation aggravated aorta endothelial cell apoptosis [52] and accelerated renal injury in diabetic rats [53]. Furthermore, the incidence rates of diabetic complications are positively correlated with the duration of diabetes [54], suggesting a continued, long-term development of the disease. Therefore, further studies are needed to clarify the effects of glucose fluctuation on PDLSCs. A long-term diabetic animal model is also necessary to explore the chronic detrimental effect of high glucose on periodontal ligament stem cells. Second, the specific mechanism of ROS upregulation and proliferation inhibition caused by high glucose was still unclear. Further experiments are needed to confirm whether MAPKs, p21^cip1^, or p16^INK4a^ are involved in the process of high glucose-induced ROS elevation and proliferation inhibition.

## 5. Conclusions

High glucose (30 mM) does not cause cell apoptosis or cell viability inhibition but exacerbates TNF-*α*-induced proliferation inhibition and cell apoptosis through upregulation and activation TNFR1.

High glucose and TNF-*α* synergistically elevate intracellular ROS levels in PDLSCs. Vitamin C at 200 *μ*M can partially restore cell viability by inhibiting intracellular ROS expression, increasing S phase entrance and mitosis.

## Data Availability

The data used to support the findings of this study are available from the corresponding author upon reasonable request.

## Supplementary Materials

Supplementary Materials Supplementary Figure S1: flow cytometry analysis of MSC surface markers in CD146+PDLCs. Second passages of CD146+PDLCs after isolation by immunomagnetic beads were detected for MSC markers expression, including (a) CD105, (b) CD90, CD73, and (c) CD34, CD11b, CD19, CD45, HLA-DR. Supplementary Figure S2: CD146-positive periodontal ligament cells exhibit characteristics of mesenchymal stem cells. (a) Colony-forming assay of CD146+PDLCs. CD146+PDLCs were seeded on culture dish (150 cells per dish) and cultured for another 14 days. Toluidine blue staining was performed to detect colonies. (Left) gross appearance; (right) cell morphology under a microscope (100x, scale bar represents 200 *μ*m). (b) Immunohistochemical staining for cytokeratin, vimentin, and Stro-1 of CD146+PDLCs. (Left) negative staining for cytokeratin (hematoxylin staining); (middle) positive staining for vimentin; (right) some cells were positive for Stro-1, whereas other cells were negative for Stro-1. Scale bar represents 200 *μ*m. (c) Osteogenic and adipogenic induction of both CD146-PDLCs and CD146+PDLCs. PDLCs were cultured under osteogenic or adipogenic conditions for 21 or 14 days; (left) mineralized nodules were detected under osteogenic conditions using alizarin red staining; (right) lipid clusters were stained with oil red O; scale bars in pictures represent 200 *μ*m. Supplementary Figure S3: influence of osmotic pressure on cell viability of PDLSCs (day 6). PDLSCs were cultured under different conditions (G5.6, G30, Mannitol, Mannitol+TNF-*α*, G5.6+TNF-*α*, and G30+TNF-*α*). For the mannitol treatment group, cells were incubated in a medium containing 5.6 mM D-glucose and 24.4 mM mannitol. Cell viability was detected by CCK-8 assay on day 6. Data are expressed as means ± standard deviations. This assay was replicated 3 times using PDLSCs obtained from 3 different individuals. ^∗^*p* < 0.05 versus the control group (G5.6), ^#^*p* < 0.05 versus the G30+TNF-*α* group. Supplementary Figure S4: detection of TNF-*α* in cell supernatant and cell lysate after treatment with high glucose and TNF-*α* on day 2. PDLSCs were treated under different conditions (G5.6, G30, G5.6+TNF-*α*, and G30+TNF-*α*) for 48 h, and ELISA was performed to detect the content of TNF-*α* (a) in the cell supernatant and (b) in the cell lysate; the value of the G5.6+TNF-*α* group was regarded as 1.0; UD denotes undetected (below the threshold value 5.6 pg/ml); ^∗^*p* < 0.01 versus the G5.6+TNF-*α* group. Supplementary Figure S5: protein expression of p-JNK and p-ERK1/2 in PDLSCs under high-glucose and TNF-*α* conditions (on day 6). PDLSCs were cultured under normal glucose or high-glucose conditions in the presence or absence of TNF-*α*. Protein expression of phosphorylated JNK and phosphorylated ERK1/2 were detected by western blotting. (a, c) Protein expression of p-JNK was elevated by TNF-*α* treatment on day 6. Data are expressed as means ± standard deviations. All assays were replicated 3 times using PDLSCs obtained from 3 different individuals. ^∗^*p* < 0.05 versus the control group. (b, d) Protein expression of p-ERK1/2 was depressed by TNF-*α* treatment on day 6, which was further inhibited under high-glucose conditions. Data are expressed as means ± standard deviations. All assays were replicated 3 times using PDLSCs obtained from 3 different individuals. ^∗^*p* < 0.05 versus the control group. ^#^*p* < 0.05 versus the G5.6+TNF-*α* group. Supplementary Figure S6: vitamin C and vitamin E partially reversed the proliferative inhibition induced by high glucose and TNF-*α*. PDLSCs were treated under different conditions (G5.6, G30, G5.6+TNF-*α*, and G30+TNF-*α*). Vitamin C (200 *μ*M) and vitamin E (200 *μ*M) were added into the culture medium 1 hour before high-glucose and TNF-*α* treatment. Cell proliferation was detected by CCK-8 assay every 24 hours. Data are expressed as means ± standard deviations. All assays were replicated 3 times using PDLSCs obtained from 3 different individuals. ^∗^*p* < 0.05 versus the control group (G5.6), ^#^*p* < 0.05 versus the G30+TNF-*α* group. △ represent the difference between the G30+TNF-*α*+Vit C group and the G30+TNF-*α*+Vit E group is statistically significant (*p* < 0.05). Supplementary Figure S7: protein expression of CDK4 in PDLSCs under high-glucose and TNF-*α* conditions (on day 6). PDLSCs were cultured under normal glucose or high-glucose conditions in the presence or absence of TNF-*α*. Protein expression of CDK4 and *β*-actin were detected by western blotting. Data are expressed as means ± standard deviations. The assay was replicated 3 times using PDLSCs obtained from 3 different individuals. ^∗∗^*p* < 0.01 versus the control group. ^#^*p* < 0.05 versus the G5.6+TNF-*α* group.
