# Supplementary material for: High Glucose Exacerbates TNF-α-Induced Proliferative Inhibition in Human Periodontal Ligament Stem Cells through Upregulation and Activation of TNF Receptor 1
Source: Stem Cells Int. 2020 Feb 5;2020:4910767. doi: 10.1155/2020/4910767 (PMC7025077; doi:10.1155/2020/4910767)
Supplement: Supplementary Materials — Supplementary Figure S1: flow cytometry analysis of MSC surface markers in CD146+PDLCs. Second passages of CD146+PDLCs after isolation by immunomagnetic beads were detected for MSC markers expression, including (a) CD105, (b) CD90, CD73, and (c) CD34, CD11b, CD19, CD45, HLA-DR. Supplementary Figure S2: CD146-positive periodontal ligament cells exhibit characteristics of mesenchymal stem cells. (a) Colony-forming assay of CD146+PDLCs. CD146+PDLCs were seeded on culture dish (150 cells per dish) and cultured for another 14 days. Toluidine blue staining was performed to detect colonies. (Left) gross appearance; (right) cell morphology under a microscope (100x, scale bar represents 200 μm). (b) Immunohistochemical staining for cytokeratin, vimentin, and Stro-1 of CD146+PDLCs. (Left) negative staining for cytokeratin (hematoxylin staining); (middle) positive staining for vimentin; (right) some cells were positive for Stro-1, whereas other cells were negative for Stro-1. Scale bar represents 200 μm. (c) Osteogenic and adipogenic induction of both CD146-PDLCs and CD146+PDLCs. PDLCs were cultured under osteogenic or adipogenic conditions for 21 or 14 days; (left) mineralized nodules were detected under osteogenic conditions using alizarin red staining; (right) lipid clusters were stained with oil red O; scale bars in pictures represent 200 μm. Supplementary Figure S3: influence of osmotic pressure on cell viability of PDLSCs (day 6). PDLSCs were cultured under different conditions (G5.6, G30, Mannitol, Mannitol+TNF-α, G5.6+TNF-α, and G30+TNF-α). For the mannitol treatment group, cells were incubated in a medium containing 5.6 mM D-glucose and 24.4 mM mannitol. Cell viability was detected by CCK-8 assay on day 6. Data are expressed as means ± standard deviations. This assay was replicated 3 times using PDLSCs obtained from 3 different individuals. ∗p < 0.05 versus the control group (G5.6), #p < 0.05 versus the G30+TNF-α group. Supplementary Figure S4: detection of TNF-α in c [file 4910767.f1.pdf]

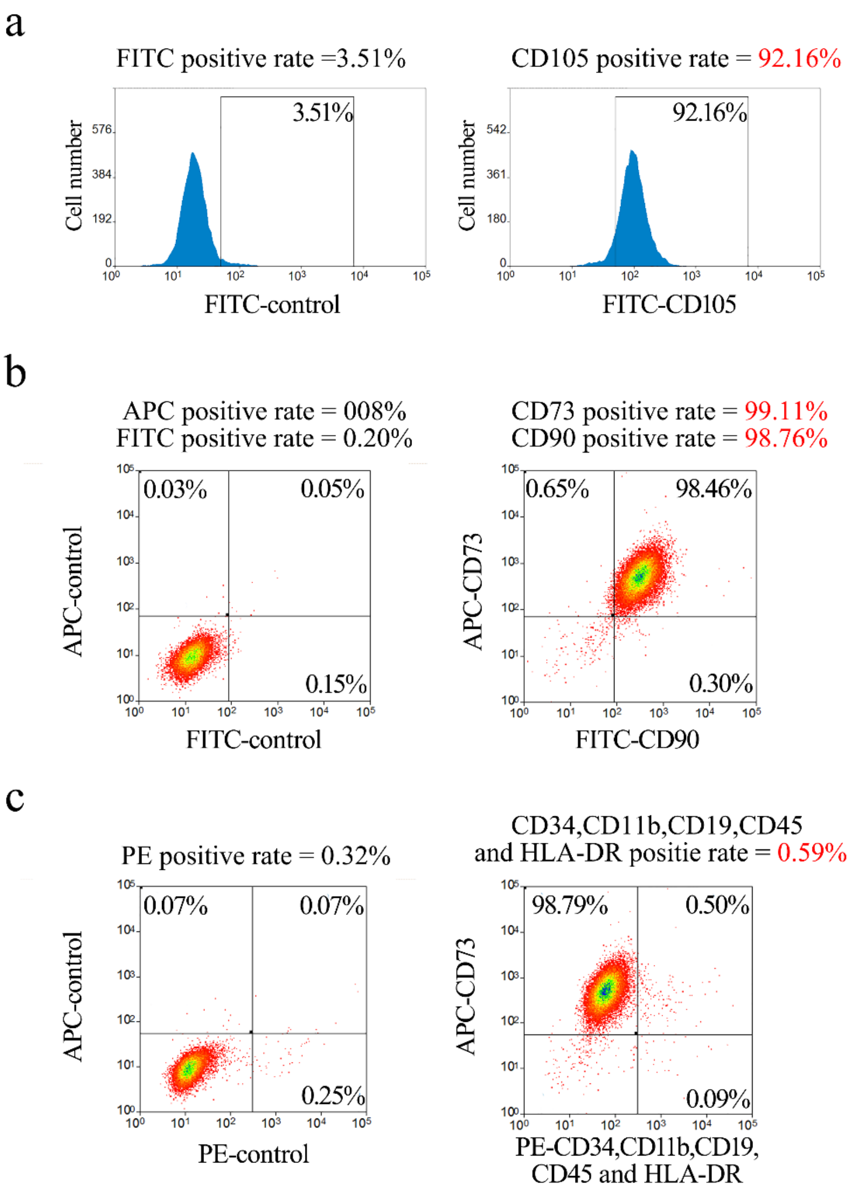

Figure S1. Flow cytometry analysis of MSC surface markers in CD146+PDLCs

Second passages of CD146+PDLCs after isolation by immunomagnetic beads were detected for MSC markers expression, including (a) CD105, (b) CD90, CD73, and (c) CD34, CD11b, CD19, CD45, HLA-DR.

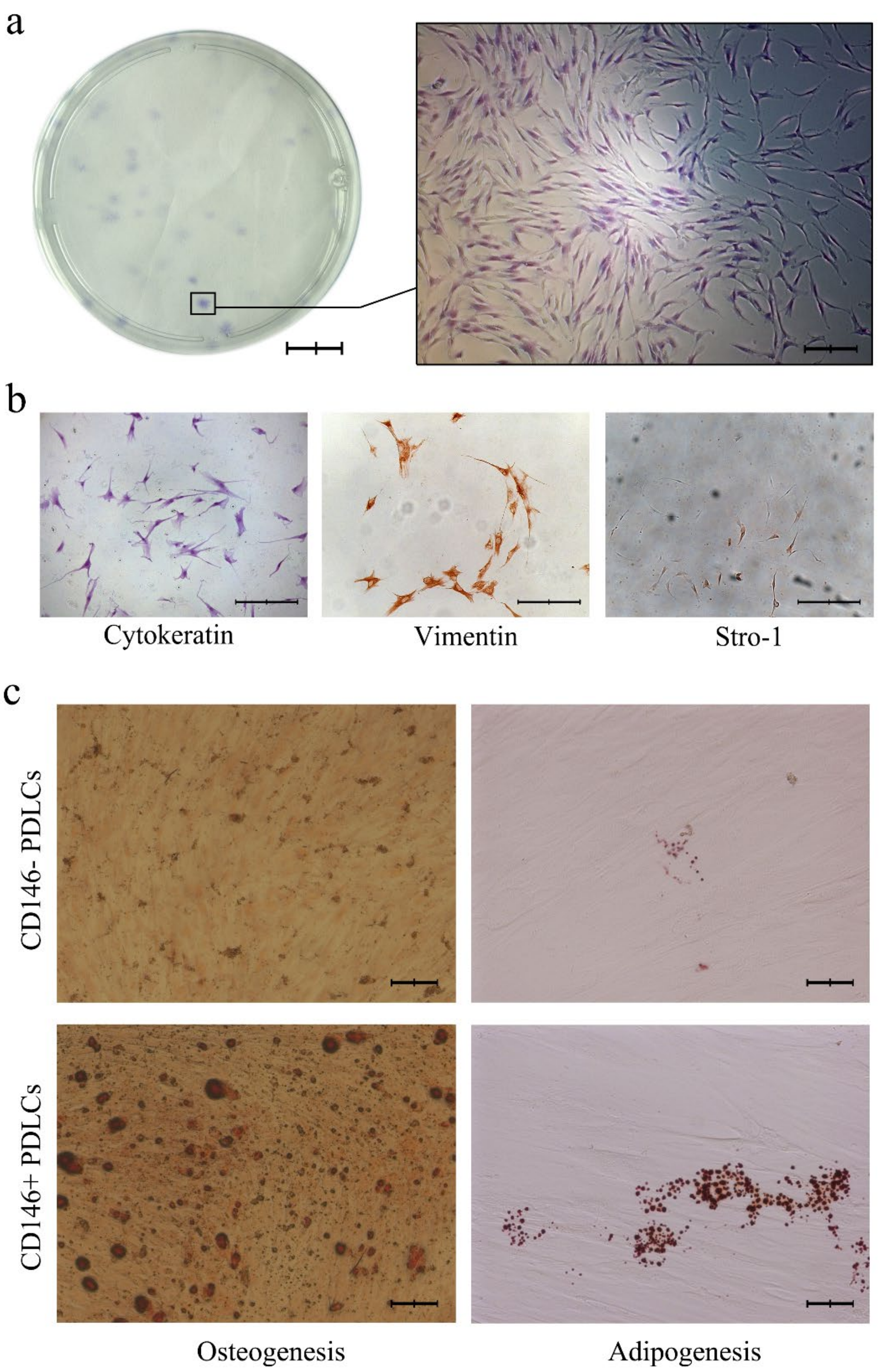

Figure S2. CD146 positive periodontal ligament cells exhibit characteristics of mesenchymal stem cells

(a) Colony-forming assay of CD146+PDLCs. CD146+PDLCs were seeded on culture dish (150 cells per dish) and cultured for another 14 days. Toluidine blue staining was performed to detect colonies. (Left) gross appearance; (Right) cell morphology under a microscope (100X, scale bar represents 200 μm).

(b) Immunohistochemical staining for cytokeratin, vimentin, and Stro-1 of CD146+PDLCs. (Left) negative staining for cytokeratin (hematoxylin staining); (Middle) positive staining for vimentin; (Right) some cells were positive for Stro-1, whereas other cells were negative for Stro-1. Scale bar represents

200μm.

(c) Osteogenic and adipogenic induction of both CD146-PDLCs and CD146+PDLCs. PDLCs were cultured under osteogenic or adipogenic conditions for 21 or 14 days; (Left) mineralized nodules were detected under osteogenic conditions using alizarin red staining; (Right) lipid clusters were stained with oil red O; Scale bars in pictures represent 200 μm.

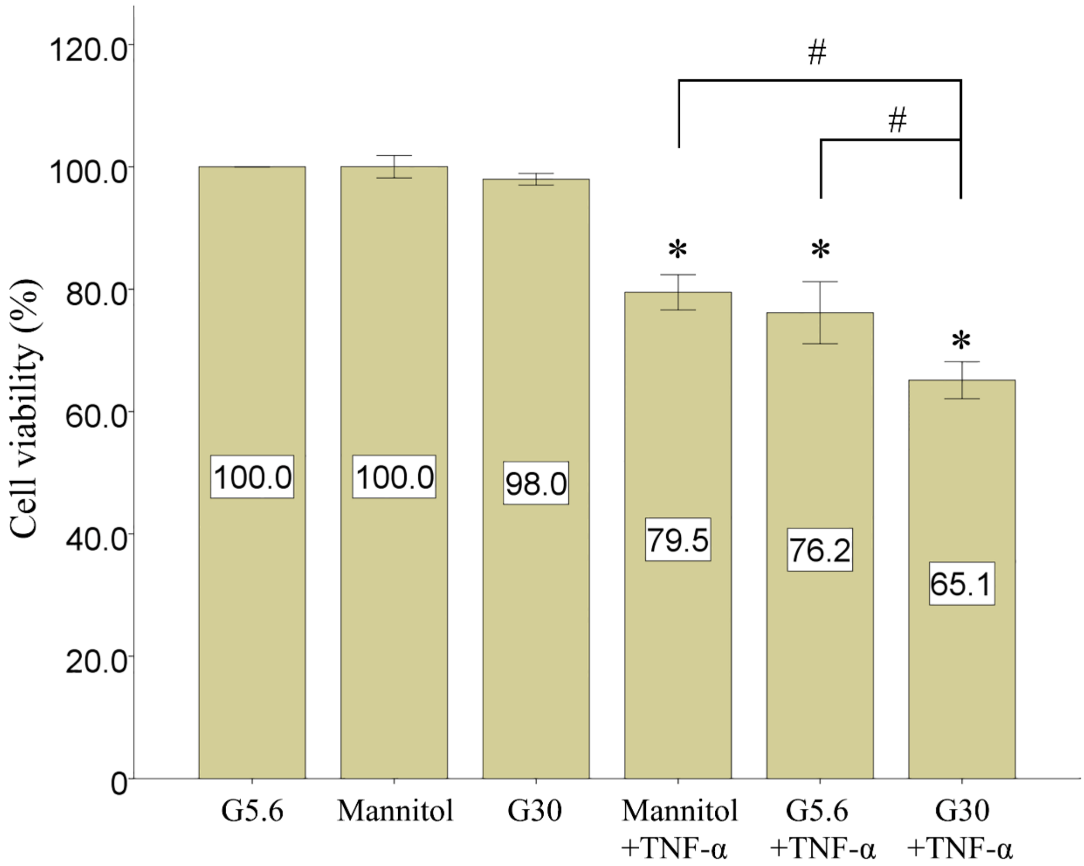

Fig S3. Influence of osmotic pressure on cell viability of PDLSCs（day 6）.

PDLSCs were cultured under different conditions (G5.6, G30, Mannitol, Mannitol+TNF-α, G5.6+TNF-α, and G30+TNF-α). For the mannitol treatment group, cells were incubated in medium containing 5.6mM D-glucose and 24.4mM mannitol. Cell viability was detected by CCK-8 assay on day 6. Data are expressed as means ± standard deviations. This assay was replicated 3 times using PDLSCs obtained from 3 different individuals. *P<0.05 versus the control group (G5.6), #p<0.05 versus G30+TNF-α group.

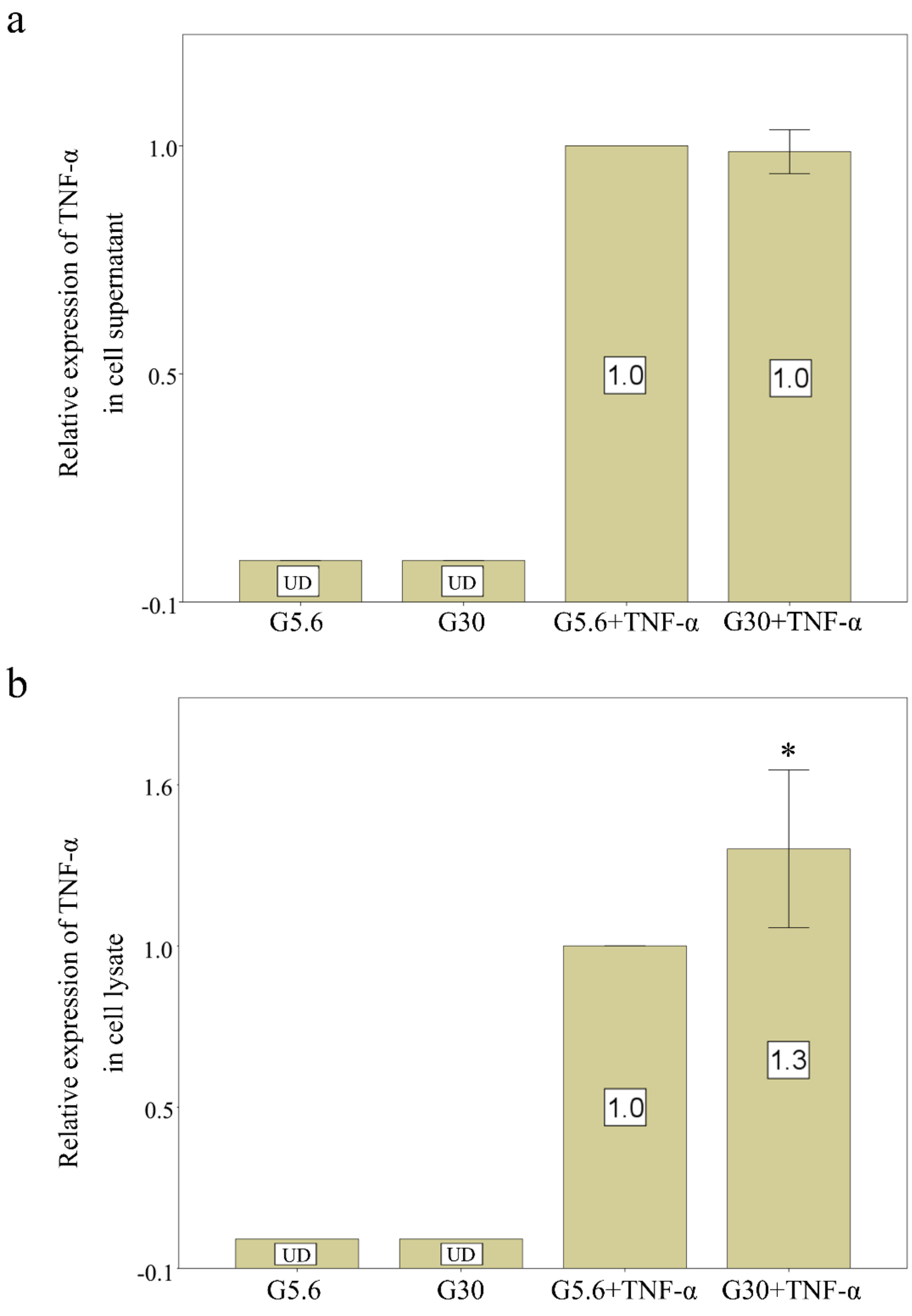

Figure S4. Detection of TNF-α in cell supernatant and cell lysate after treatment with high glucose and TNF-α on day 2

PDLSCs were treated under different conditions (G5.6, G30, G5.6+TNF-α, and G30+TNF-α) for 48h, and ELISA was performed to detect the content of TNF-α (a) in the cell supernatant and (b) in the cell lysate; The value of G5.6+TNF-α group was regarded as 1.0; UD denotes undetected (below the threshold value 5.6 pg/ml of the ELISA kit); *$p<0.01$ versus G5.6+TNF-α group

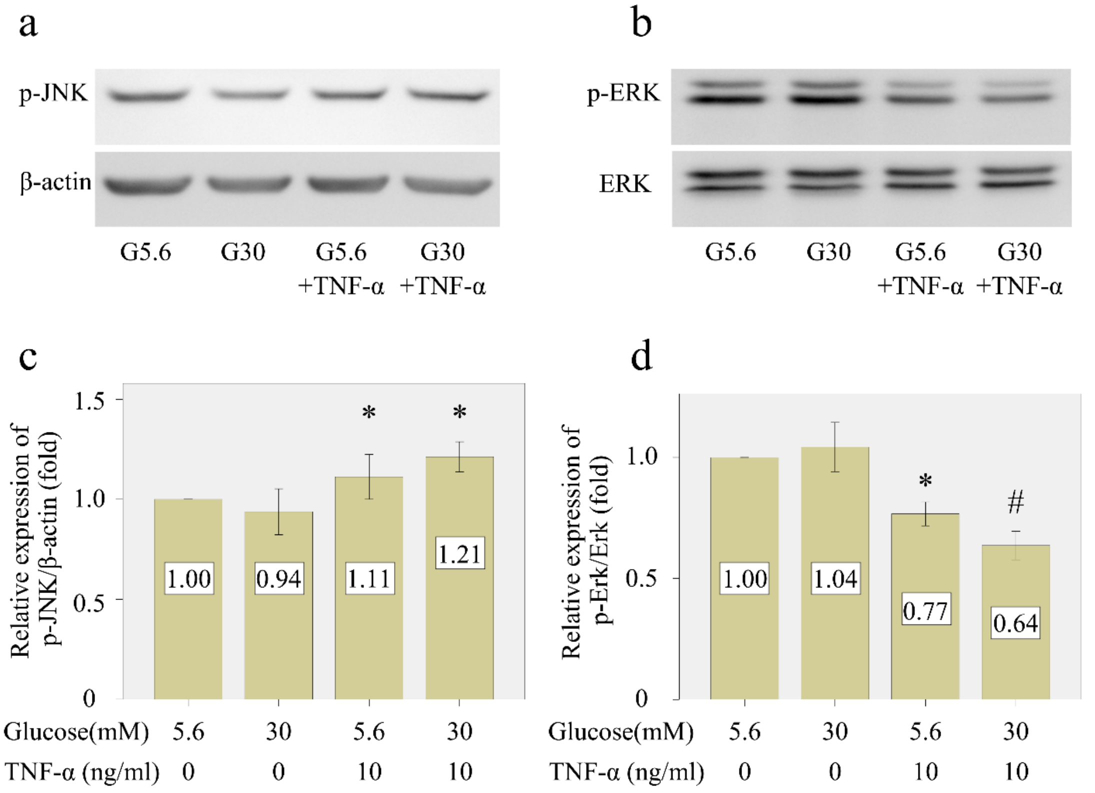

Figure S5. Protein expression of p-JNK and p-ERK1/2 in PDLSCs under high glucose and TNF-α conditions (on day 6)

PDLSCs were cultured under normal glucose or high glucose conditions in the presence or absence of TNF-α. Protein expression of phosphorylated JNK and phosphorylated ERK1/2 were detected by western blotting.

(a and c) Protein expression of p-JNK was elevated by TNF-α treatment on day 6. Data are expressed as means ± standard deviations. All assays were replicated 3 times using PDLSCs obtained from 3 different individuals. *$p<0.05$ versus the control group.

(b and d) Protein expression of p-ERK1/2 was depressed by TNF-α treatment on day 6, which was further inhibited under high glucose conditions. Data are expressed as means ± standard deviations. All assays were replicated 3 times using PDLSCs obtained from 3 different individuals. *$p<0.05$ versus the control group. #$p<0.05$ versus G5.6+TNF-α group.

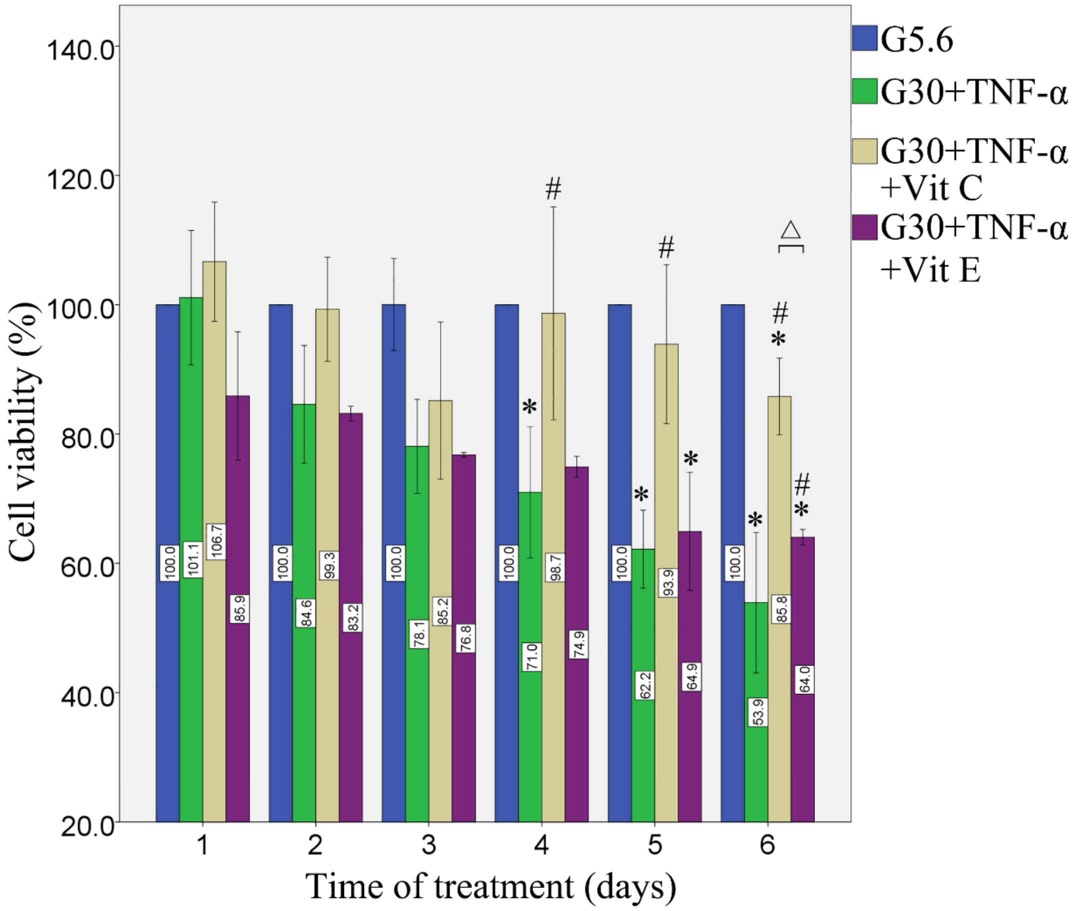

Fig S6. Vitamin C and vitamin E partially reversed the proliferative inhibition induced by high glucose and TNF-α.

PDLSCs were treated under different conditions (G5.6、G30、G5.6+TNF-α、G30+TNF-α). Vitamin C (200μM) and vitamin E (200μM) was added into culture medium 1 hour before high glucose and TNF-α treatment. Cell proliferation was detected by CCK-8 assay every 24 hours. Data are expressed as means ± standard deviations. All assays were replicated 3 times using PDLSCs obtained from 3 different individuals. *P<0.05 versus the control group (G5.6), #p<0.05 versus G30+TNF-α group. △ represent the difference between the G30+TNF-α+Vit C group and the G30+TNF-α+Vit E group is statistically significant (p<0.05).

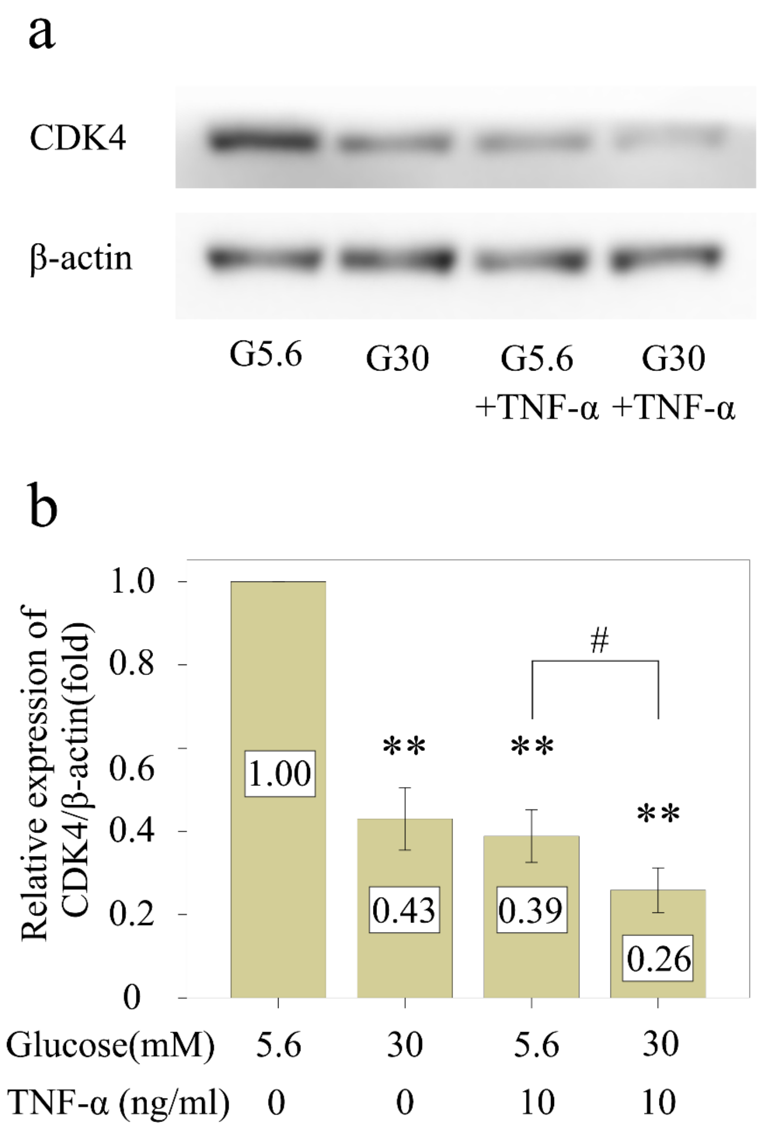

Figure S7. Protein expression of CDK4 in PDLSCs under high glucose and TNF-α conditions (on day 6)

PDLSCs were cultured under normal glucose or high glucose conditions in the presence or absence of TNF-α. Protein expression of CDK4 and β-actin were detected by western blotting. Data are expressed as means ± standard deviations. The assay was replicated 3 times using PDLSCs obtained from 3 different individuals. **p<0.01 versus the control group. #p<0.05 versus G5.6+TNF-α group.
